# Atypical/malignant solitary fibrous tumor of the pancreas with spleen vein invasion: Case report and literature review

**DOI:** 10.1097/MD.0000000000019783

**Published:** 2020-04-24

**Authors:** Jingdong Li, Jiangpeng Li, Yongfu Xiong, Ting Xu, Jian Xu, Qiang Li, Gang Yang

**Affiliations:** a Department of Hepatobiliary Surgery, Affiliated Hospital of North Sichuan Medical College; b Institute of Hepato-Biliary-Intestinal Disease, North Sichuan Medical College; c Department of Emergency, Affiliated Hospital of North Sichuan Medical College, Nanchong, China.

**Keywords:** case report, immunohistochemistry, pancreas, solitary fibrous tumor, surgical treatment

## Abstract

**Introduction:**

Solitary fibrous tumor (SFT) is an uncommon mesenchymal tumor that is most common in the pleura. However, according to previous studies, the SFT of the pancreas is extremely rare; only 20 cases have been reported so far. Here, we conduct a literature review and report the first case of atypical/malignant SFT of the pancreas with spleen vein invasion.

**Patient concerns:**

The patient is a 61-year-old Chinese male who presented with 1 week of upper abdominal pain. Abdominal magnetic resonance imaging showed a huge mass (>10 cm) at the distal end of the pancreas, and the mass obstructing the splenic vein.

**Diagnosis:**

Atypical/malignant SFT of the pancreas with splenic vein tumor thrombus.

**Interventions:**

The patient underwent laparoscopic distal pancreatectomy with splenectomy procedure to achieve a radical resection, and did not undergo chemotherapy or radiotherapy.

**Outcomes:**

Abdominal computed tomography scans were performed at 1 and 4 months after resection, and no signs of recurrence or metastasis were found (Fig. 1

**Figure 1:**
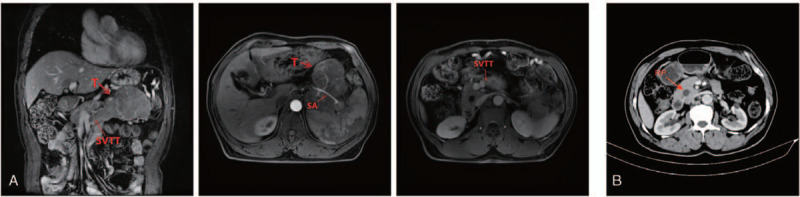
MRI of the abdomen. (A) Preoperative MRI: The mass at the distal end of the pancreas is shown by an axial view and a coronal view. (B) Postoperative MRI: no signs of recurrence were found. RP = residual pancreas, SA = splenic artery, SV = splenic vein, SVTT = splenic vein tumor thrombus, T = Tumor.

. B).

**Conclusion:**

The clinical symptoms of atypical/malignant SFT of the pancreas with spleen vein invasion are not atypical, and imaging feature is lack of specificity. Preoperative diagnosis is difficult, and there is a potential for malignancy. However, due to the paucity of randomized control trials, there is no established, globally accepted treatment strategy, radiation therapy and chemotherapy regimens have not demonstrated global effectiveness, and no standardized treatments have been identified. Therefore, we recommend complete surgical resection and close clinical follow-up.

## 1 Introduction

Solitary fibrous tumor (SFT) is a rare spindle cell tumor that was first described at the pleura.^[1]^ It is found in almost any part of the body.^[2]^ And SFT from the pancreas is extremely rare; only 20 cases have been reported since 1999 (Table 1). There is no gender difference in this tumor, which is mainly observed in the middle-aged individuals. Most tumors show benign histology without splenic vein invasion, but about 12% to 22% of cases show aggressive behavior, such as local recurrence and metastasis.^[3]^ Assessment of metastatic risk is a source of debate. We lack consensus on the diagnosis and treatment of this disease. In consideration of small numbers, data about recurrence or survival are not that accurate and no established treatment modality or follow-up plan has been agreed on. Therefore, it is necessary to conduct a detailed review of the existing literature to fully understand the diagnosis and treatment status of SFT.

**Table 1:**
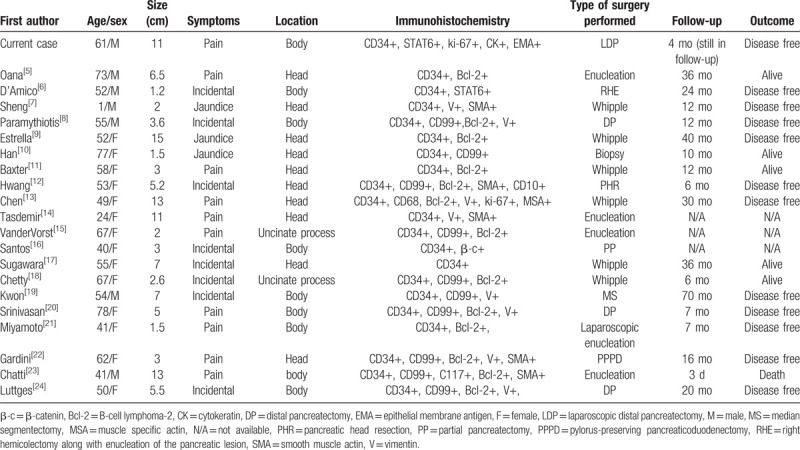
Main characteristics of reported pancreas solitary fibrous tumor cases.

## 2 Case report

A 61-year-old male patient was admitted to the hospital because of pain in the upper abdomen for 1 week. This patient had no discomfort such as nausea, vomiting, chills, fever, bloating, diarrhea, anorexia, fatigue, blood in the stool, and itching of the skin. Apart from meningioma operation that was performed 20 years before, no other surgical history was present. He also had no history of hypertension, diabetes, or heart disease. He denied any history of smoking, alcohol, and drug abuse. The physical examination showed that the whole abdomen was soft, no tenderness, rebound tenderness and muscle tension, the upper abdomen could touch a mass of about 4.0 × 2.0 cm, its texture was hard, and the activity was very poor. Furthermore, results of all laboratory tests including complete blood counting, biochemistry, blood coagulation test, and tumor markers such as CAl99, CA50, CA242, and CEA were within normal limits. Magnetic resource imaging (MRI) of abdomen demonstrated a 10 cm lobulated mass at the distal end of the pancreas, and there was closed relationship between the mass and the stomach, splenic artery, and splenic vein (Fig. 1, A). It is not difficult to see the mass obstructing the splenic vein (Fig. 1, A, splenic vein tumor thrombus). The mass showed a low signal on the T1-weighted MRI and a high signal on the T2-weighted MRI. Magnetic resonance cholangiopancreatography showed no expansion of the main pancreatic duct. Regional lymphadenopathy and distant metastasis (liver, brain, lung, bone, bone marrow) was not noted. Preoperatively, we treat the lesion as pancreatic cancer or gastrointestinal stromal tumor with invasion of the splenic vein.

**Figure 2:**
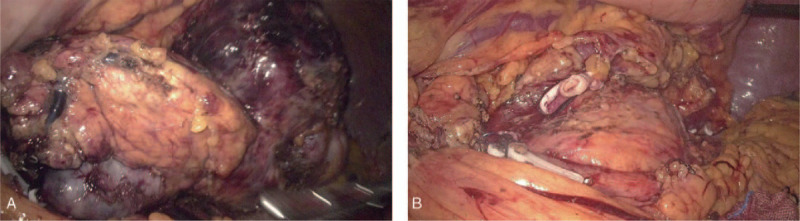
Intra-operative appearance of the pancreatic mass. (A) Laparoscopic surgery shows a mass, approximately 10.0 cm in greatest dimension, near the distal of the pancreas (T). (B) Surgical area display after laparoscopic distal pancreatectomy and splenectomy.

**Figure 3:**
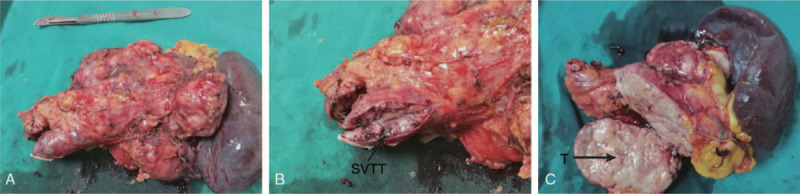
(A) Complete specimen. (B) Tumor thrombus visible in the splenic vein. (C) A macroscopic examination revealed a 13 × 11 × 10 cm tumor that was oval, lobulate, well circumscribed, and well-capsulated firm mass; the cut surface was yellowish-gray, a fish flesh. SVTT = splenic vein tumor thrombus, T = tumor.

**Figure 4:**
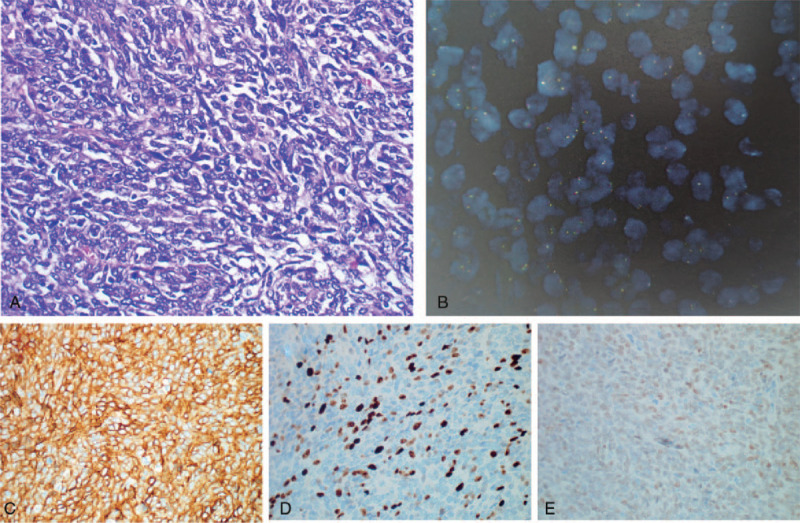
Immunohistochemical results of solitary fibrous tumor of the pancreas. A: A microscopic examination showed patternless spindle cell neoplastic proliferation, HE stain, magnification: ×400, nuclear division is about 25/10 HPF; B: The SSX/SS18 gene ectopic was not detected by fluorescence in situ hybridization; C, D, and E: Immunohistochemical: CD34+, STAT6+, ki-67+. Magnification: ×400. HE = hematoxylin and eosin, HPF = high-power field.

For further diagnosis and treatment, laparoscopic distal pancreatectomy with splenectomy was performed with no complications (Fig. 2). A macroscopic examination revealed a 13 × 11 × 10 cm tumor that was oval, lobulate, well circumscribed, and well-capsulated firm mass. The cut surface was yellowish-gray, a fish flesh, and tumor invasion visible in the splenic vein (Fig. 3, B, splenic vein tumor thrombus). Hematoxylin-eosin staining images of excised specimen showed that the tumor contained spindle cells, and some atypical mitotic figures (25 per 10 high-power fields [HPFs]) were also noted in the hypercellular area (Fig. 4, A). Immunohistochemically (Fig. 4, C–E), this tumor tested positive for CD34, STAT-6, cytokeratin (focality), epithelial membrane antigen (focality), and Ki-67 (20%) and negative for Desmin, Dog1, S-100, TLE-1, β-catenin (β-c), CD21, CD35, CD23, and CD117.^[4]^ In addition, fluorescence in situ hybridization also did not detect SSX/SS18 gene ectopic (Fig. 4, B). Therefore, this patient was diagnosed as having atypical/malignant SFT of the pancreas with spleen vein invasion. According to the 2013 WHO classification of soft tissue tumors, SFT is now considered as a fibroblastic/myofibroblastic tumor.^[5]^ And the American Joint Committee on Cancer staging system is the most commonly used staging system for soft tissue sarcomas; this system takes into account characteristics of the primary tumor (T), lymph nodes (N), distant metastasis (M), and grade (G).

Finally, this patient was discharged on day 8 after surgery and did not undergo chemotherapy or radiotherapy. Then, abdominal computed tomography (CT) scans were performed at 1 and 4 months after resection, and no signs of recurrence or metastasis were found (Fig. 1. B).

## 3 Discussion

SFT was first described as a unique mesenchymal tumor in 1931 at pleura; it occasionally develops in extra pleural locations.^[1]^ On PubMed (https://www.ncbi.nlm.nih.gov/pubmed/) and Google (https://www.google.com/), we performed a literature search using the following combination of keywords: pancreas and solitary fibrous tumor. We have summarized all available cases, and only 20 cases of SFT have occurred in the pancreas in the literature since 1999 (Table 1).^[6–25]^ They are almost case reports.

After summarizing and analyzing all the cases, we found that SFT in the pancreas occurs slightly more frequently in women (14/21, 66.66%) than in men, with a median age of 54 (range 1–78). Tumor predominantly occurred in the head (12/21, 57.14%) of pancreas, and the median tumor size was 5.0 cm (range 1.2–15.0 cm). Regarding clinical manifestations, the main symptoms were abdominal pain (10/21, 47.62%) and jaundice (3/21, 14.30%), and the rest of the tumors were found incidentally (8/21, 38.10%). Regarding imaging, all tumors showed an enhancement by arterial and portal CT, and 7 cases (including our study) described the results of MRI imaging: these tumors showed low signals in T1-weighted images and high signals in T2-weighted images.^[9,11,13,18,20,24]^ However, diagnosis based on imaging results is generally considered to be endocrine neoplasms.^[19,21]^

As the clinical symptoms of SFT in the pancreas are not typical, and they are also difficult to distinguish radiologically, it is very difficult to diagnose such diseases before surgery. In addition, in this position, it may be misdiagnosed as other more common tumors, such as pancreatic cancer, gastrointestinal stromal tumor,^[4]^ schwannoma, liposarcoma, leiomyosarcoma, and islet cell tumor.^[17]^ Therefore, we have always believed that the combination of histopathological analysis and immunohistochemistry is the gold standard for diagnosing an SFT. In particular, the immunochemistry can accurately distinguish between SFT and other mesenchymal tumors.^[26]^

Grossly, histological examination revealed that SFT usually consists of spindle-shaped tumor cells arranged in a “modeless pattern,” and nuclear polymorphism is mild to moderate, with mitotic rates ranging from 0/10 to 30/10 HPF. Numerous studies have shown that nuclear atypia, high mitotic count (>4/10 HPF), tumor sizes, tumor sites, positive surgical margins, and necrosis are the most useful malignant indicators.^[10,27,28]^ On immunohistochemical study, CD34, CD99, and Bcl-2 were previously thought to be the most useful positive immunohistochemical markers for SFT. But, the latest research shows that STAT6 is an almost completely specific and highly sensitive immunohistochemical marker for SFT.^[29,30]^ In addition, molecular genetics identified the fusion of NAB2-STAT6 as the latest molecular marker, with high sensitivity and specificity.^[1,27]^ According to morphology, histological, and immunohistochemistry results, it is clear that our patients meet the diagnostic criteria for atypical/malignant SFT of the pancreas with spleen vein invasion.

About treatment, due to the paucity of randomized control trials, there is no established, globally accepted treatment strategy. In all our cases, most of SFT show benign histology (19/21, 90.49%) and have no spleen vein invasion. And all patients, but 1,^[11]^ have been surgically treated. Whipple is the most frequently performed surgical procedure (7 cases), followed by tumor enucleation procedure (6 cases), distal pancreatectomy (4 cases), segmental pancreatic resection (3 cases), and laparoscopic distal pancreatectomy (1 cases). Among them, the patient from Chatti et al^[24]^ died of surgical complications on the third postoperative day, and the remaining patients survived with a median follow-up of 12 months. It is worth noting that the patient from Sugawara et al^[18]^ survived for 36 months after surgery; the patient from Kwon et al^[20]^ was still alive 70 months after surgery and showed no signs of recurrence; even the patient with malignant SFT diagnosis at pathology is still alive with no evidence of disease at 40 months.^[10]^ Like many other soft tissue tumors, surgical management is the mainstay of treatment for SFT with emphasis on obtaining tumor negative margins.^[1]^ And, obtaining adequate negative margins has been shown to decrease the rate local disease recurrence and improve survival.^[31]^

Because it is the first case of atypical/malignant SFT of the pancreas with spleen vein invasion, the risk of metastasis has not been evaluated to date. And based on the above data, we believe that radical surgical resection remains the gold standard for treatment, even in malignant cases with splenic vein invasion approved by histopathology. Besides, radiation therapy and chemotherapy regimens have not demonstrated global effectiveness, and no standardized treatments have been identified.^[1]^ Therefore, the patient underwent laparoscopic distal pancreatectomy with splenectomy procedure to achieve a radical resection, and did not undergo chemotherapy or radiotherapy. Fortunately, the patients in this study underwent abdominal CT scans at 1 and 4 months after resection, and no signs of recurrence or metastasis were found (Fig. 1. B). But it is still necessary to continue follow-up.

## 4 Conclusion

In summary, SFT of the pancreas with splenic vein invasion is extremely rare, clinical symptoms are not typical, and imaging feature is lack of specificity. This poses a huge challenge to preoperative diagnosis and is likely to be missed or misdiagnosed. Therefore, when confronted with pancreatic tumors, the possibility of SFT should be considered and confirmed by immunohistochemistry. On the other hand, it is a consensus that surgical resection and long-term follow-up are the most suitable treatments for SFT, but radiation and chemotherapy may have potential roles in the treatment algorithm. Although several challenges are faced in terms of treatment, we believe that our results might be useful in future studies of such disease.

## Author contributions

**Jingdong Li:** constructed the idea of the case and revised the manuscript critically for important intellectual content and approved it for publication

**Jiangpeng Li:** constructed the idea of the case, reviewed the literature, and designed the manuscript

**Jingdong Li and Jiangpeng Li:** composed the manuscript and literature review

**Supervision:** Yongfu Xiong.

**Resources:** Ting Xu.

**Writing – original draft:** Jiangpeng Li.

**Writing – review & editing:** Jingdong Li, Yongfu Xiong, Jian Xu, Qiang Li, Gang Yang.

Jingdong Li: ORCID 0000-0002-1923-1787.

Jingdong Li orcid: 0000-0002-1923-1787.
